# Clinical and Laboratory Characteristics of Kikuchi-Fujimoto Disease According to Age

**DOI:** 10.3389/fped.2021.745506

**Published:** 2021-11-02

**Authors:** Hye-Young Kim, Ha Young Jo, Seong Heon Kim

**Affiliations:** ^1^Department of Pediatrics, Medical Research Institute, Pusan National University School of Medicine, Pusan National University Hospital, Busan, South Korea; ^2^Department of Pediatrics, Pusan National University Hospital, Busan, South Korea; ^3^Department of Pediatrics, Seoul National University Children's Hospital & College of Medicine, Seoul, South Korea

**Keywords:** Kikuchi-Fujimoto disease, histiocytic necrotizing lymphadenitis, prognosis, children, adults, age

## Abstract

**Background:** Little information exists regarding the differences in the clinical and laboratory characteristics of Kikuchi-Fujimoto disease (KFD) according to age.

**Objective:** To evaluate the clinical and laboratory characteristics of KFD according to age.

**Methods:** The relevance of sex, age, clinical features, laboratory findings, courses, and follow-up results were retrospectively evaluated in patients diagnosed with KFD at Pusan National University Hospital between 2010 and 2020.

**Results:** Eighty patients (46 children and 34 adults) with a mean age of 21.5 ± 11.8 years (range, 3–49 years) were included in the study. Those aged 10–19 years accounted for the largest number of patients (42.5%). Among children, the male sex ratio was higher, especially for patients aged ≤ 9 years. In adults, the female sex ratio was higher, especially for patients aged 20–29 years. Fever, tenderness in the lymph node, and skin rashes were more common in children, while myalgia and weight loss were more common in adults. In children, the recurrence rate was significantly higher among boys than among girls (15.8 vs. 0.0%, *P* = 0.001); lower platelet count and higher CRP levels were observed among boys than among girls. EBV and ANA positivity rates were higher in boys than in girls. In adults, the recurrence rate was significantly higher in women than in men (18.2 vs. 0.0%, *P* = 0.005). ANA positivity rates were higher in women than in men.

**Conclusion:** The clinical features, laboratory findings, and recurrence of KFD may differ depending on age and sex. Clinicians should be aware of this.

## Introduction

Kikuchi-Fujimoto disease (KFD), also called Kikuchi disease or histiocytic necrotizing lymphadenitis, is a rare, generally self-limiting condition of unknown cause, usually characterized by cervical lymphadenopathy, fever, and leukopenia (1). As the symptoms are non-specific, differential diagnoses, including viral infections, malignancies, and autoimmune conditions such as systemic lupus erythematosus (SLE), are often considered. The diagnosis of KFD is usually based on lymph node histology, which features variably confluent paracortical necrosis surrounded by a prominent collar of histiocytes with crescentic nuclei, immunoblasts, and plasmacytoid monocytes.

While the pathogenesis of Kikuchi disease is unknown, the clinical presentation, course, and histologic changes suggest an immune response of T cells and histiocytes to an infectious agent. Infectious agents, including Yersinia, Toxoplasma, Epstein–Barr virus, human herpesvirus 6 and 8, human T-lymphotropic virus type 1, and parvovirus B19 have been reported to play a causative role, but this has not been confirmed (2, 3).

Although KFD affects all age and sex groups, the clinical features of KFD may differ according to age and sex. It was primarily thought to be a disease affecting women under the age of 30 years. However, in a Korean report of 20 individuals younger than 18 years of age with Kikuchi disease, the sex distribution is equal (4). Others have suggested that, among children, boys are slightly more frequently affected than girls, in contrast to older patients (5, 6). In addition, KFD has a reported recurrence rate of 3–4% (7), but pediatric studies have shown a higher recurrence rate of up to 42.4% (8–10).

The age- and sex-related differences in KFD characteristics are not yet fully understood. Many KFD studies have focused on adults; thus, there is little information on the differences in the presentation of KFD according to age. Therefore, it is necessary to understand the differences in the clinical and laboratory features of KFD according to age and sex. We analyzed the clinical and laboratory characteristics of patients with KFD according to age and sex.

## Materials and Methods

We reviewed the medical records of KFD patients who were diagnosed between January 2010 and September 2020 at Pusan National University Hospital. Our university hospital is a reference center for six million inhabitants in Busan and Gyeongnam, Korea. The study protocol was approved by the institutional review board of Pusan National University Hospital (PNUHIRB 2012-032-098). The diagnosis of KFD was made on the basis of histopathologic findings of affected lymph nodes obtained by fine-needle aspiration (FNA) or excisional biopsy after exclusion of other diseases, such as multifocal necrosis in the paracortical area with karyorrhectic debris and various histiocytes with crescentic nuclei in the absence of neutrophils (11–13). Most of our pathology results were as follows: (1) Karryorhectic debris, necrosis, and histiocytes, suspicious for histiocytic necrotizing lymphadenitis (Kikuchi-Fujimoto disease) (2) Histiocytic necrotizing lymphadenitis, consistent with Kikuchi disease. Immunohistochemistry of CD3, CD20, and Ki67 for the lymphoid tumor was done and revealed mostly reactive patterns. Unfortunately, we did not perform the Epstein-Barr virus (EBV)-encoded RNA *in-situ* hybridization.

Initially, FNA was performed in 68 patients, and an excisional biopsy was performed in 12 patients. FNA cytology was used as a diagnostic test in 61 of 68 patients, and the remaining seven patients required an additional excisional biopsy. The decision on the performance and timing of the histopathologic confirmation was made depending on the patient's condition and the severity and duration of their symptom manifestations. Clinical features, laboratory findings, courses, and follow-up results were collected and analyzed. Subjects under 19 years of age were defined as children, and those above or equal to 19 years of age were defined as adults. EBV positivity was defined when the EBV IgM viral capsid antigen (VCA) or EBV polymerase chain reaction (PCR) blood assays were positive.

Statistical analysis was performed using SPSS for Windows (version 21.0; SPSS, Chicago, IL, USA). The data are expressed as means ± standard deviations or as percentages where appropriate. The clinical and demographic data were compared between groups using the chi-square analysis and the Mann-Whitney *U*-test. *P* < 0.05 were considered statistically significant.

## Results

A total of 80 patients (46 children and 34 adults) were included in the study. Twenty (43.5%) children and 22 (64.7%) adults were female, and the proportion of female patients was significantly higher among adults than among children (*P* = 0.003) (Table 1). The mean age of the study subjects was 21.5 ± 11.8 years (range, 3–49 years). The mean age was 13.2 ± 4.8 years for children and 32.7 ± 8.8 years for adults. Among the 80 patients, the age distribution was as follows: 12 were (15.0%) aged ≤ 9, 34 (42.5%) were aged 10–19, 14 (17.5%) were aged 20–29, 14 (17.5%) were aged 30–39, and 6 (7.5%) were aged ≥ 40 years. Those aged between 10 and 19 years accounted for the largest number of patients (42.5%). Two children and one adult had a positive family history of autoimmune diseases, all of which were SLE.

**Table 1:** Manifestations of Kikuchi-Fujimoto disease by age.

**Characteristics**	**Total**	**Children**	**Adults**	***P*-value**
Number of patients	80	46	34	
Sex (Male/female)	38/42	26/20	12/22	0.003
Age group (years)	21.5 ± 11.8	13.2 ± 4.8	32.7 ± 8.8	
0–9	12 (15.0)			
10–19	34 (42.5)			
20–29	14 (17.5)			
30–39	14 (17.5)			
≥40	6 (7.5)			
Positive family history of autoimmune disease	3 (3.8)	2 (4.3)	1 (2.9)	1.0
Symptoms				
Fever	52 (65.0)	34 (73.9)	18 (52.9)	0.005
Tender lymph node	48 (60.0)	34 (73.9)	14 (41.2)	0.002
Myalgia	16 (20.0)	6 (13.0)	10 (29.4)	0.013
Weight loss (>2 kg)	10 (12.5)	4 (8.7)	6 (17.6)	0.012
Arthralgia	8 (10.0)	4 (8.7)	4 (11.8)	0.062
Rash	6 (7.5)	6 (13.0)	0 (0.0)	0.001
Lymphadenopathy				
Cervical (%)	72 (90.0)	44 (95.7)	28 (82.4)	0.023
Unilateral (%)	44 (55.0)	26 (56.5)	18 (52.9)	0.121
Bilateral (%)	28 (35.0)	18 (39.1)	10 (29.4)	0.021
Generalized (%)	8 (10.0)	2 (4.3)	6 (17.6)	0.015
LN max. diameter (cm)	1.93 ± 1.09	2.35 ± 0.57	1.93 ± 0.46	0.078
Relapse	8 (10.0)	4 (8.7)	4 (11.8)	0.107
Laboratory findings				
WBC (/mm^3^)	4,234 ± 1,761	4,165 ± 1,366	4,333 ± 2,264	0.214
ANC (/mm^3^)	2,413 ± 1,475	2,110 ± 1,017	2,845 ± 1,916	0.099
Hb (mg/dL)	12.8 ± 3.7	12.9 ± 2.4	12.7 ± 4.1	0.453
Platelet (/mm^3^)	256 ± 66	266 ± 71	251 ± 89	0.667
LDH (IU/l)	477 ± 338	509 ± 340	416 ± 349	0.327
AST (IU/l)	49 ± 41	53 ± 46	42 ± 33	0.201
ALT (IU/l)	40 ± 58	42 ± 45	37 ± 74	0.222
CRP (mg/dl)	1.30 ± 2.04	0.93 ± 0.98	1.82 ± 2.94	0.078
ESR (mm/hr)	34.8 ± 23.4	32.7 ± 20.1	37.1 ± 26.4	0.122
EBV positive, n/N (%)	8/48 (16.7)	8/28 (28.5)	0/20 (0.0)	0.001
ANA positive, n/N (%)	15/55 (27.3)	8/30 (26.6)	7/22 (31.8)	0.098
Homogeneous	4/15	2/8	2/7	
Speckled	4/15	3/8	1/7	
Nuclear	4/15	2/8	2/7	
Cytoplasmic	3/15	1/8	2/7	
Low C3 or C4	0/15	0/8	0/7	
Positive ds DNA Ab	0/15	0/8	0/7	
Positive other autoantibodies (smith, Ro, La)	0/10	0/4	0/6	

*N indicates the total number of patients who underwent the test*.

*Data are shown as mean ± standard deviation or median with interquartile range or percentage. LN, lymph node; WBC, white blood cell; ANC, absolute neutrophil; Hb, hemoglobin; LDH, lactate dehydrogenase; AST, aspartate transaminase; ALT, alanine transaminase; CRP, C-reactive protein; ESR, erythrocyte sedimentation rate; EBV, Epstein-barr virus; ANA, anti-nuclear antibodies*.

Fifty-two patients (65.0%) had a fever, and tender lymph nodes were observed in 60.0% of patients. Fever and tender lymph nodes were far more prevalent in children than in adults (73.9 vs. 52.9%, *P* = 0.005; 73.9 vs. 41.2%, *P* = 0.002, respectively). Myalgia and weight loss (>2 kg) occurred in 13.0 and 8.7% of the children and in 29.4 and 17.6% of the adults, respectively. These symptoms were significantly higher in adults than in children (*P* = 0.013 and *P* = 0.012, respectively). Although rash was not common, it was more common in children (13.0 vs. 0%, *P* = 0.001). Most skin lesions in children were like non-specific erythematous maculopapular rashes, mainly on the face trunk and upper extremities accompanied by mild itching.

All patients presented with lymphadenopathy, with cervical nodes involved in 72 (90.0%) patients, axillary nodes involved in 7 (8.8%) patients, inguinal nodes involved in 5 (6.3%), mesenteric nodes involved in 2 (2.5%), and generalized lymphadenopathy involving two or more anatomic sites in eight (10.0%) patients. While cervical lymphadenopathy, especially bilateral lymphadenopathy, was more common in children than in adults, generalized lymphadenopathy was more common in adults than in children (Table 1). There was no difference in lymph node size between the children and adults. There were four recurrent cases in children and adults, respectively. The rate of EBV positivity was higher in children than in adults (28.5 vs. 0.0%, *P* = 0.001). The other laboratory findings, including WBC, Hb, platelet, AST ALT, C-reactive protein (CRP), ESR, and antinuclear antibody (ANA) positivity rates, were not different between the two groups (Table 1).

In children, the male sex ratio was higher, especially for patients aged ≤ 9 years (Table 2). Fever was more common in boys than in girls (92.3 vs. 50.0%, *P* = 0.002). Bilateral cervical lymphadenopathy was observed more frequently in boys than in girls (53.8 vs. 20.0%, *P* = 0.001). The recurrence rate was also significantly higher in boys than in girls (15.8 vs. 0.0%, *P* = 0.001). In boys, we found lower platelet counts and higher CRP levels than in girls. Furthermore, EBV and ANA positivity rates were higher in boys than in girls (Table 2).

**Table 2:** Manifestations of Kikuchi-Fujimoto disease in children by sex.

**Characteristics**	**Total**	**Male**	**Female**	***P*-value**
Number of patients (%)	46 (100)	26 (56.5)	20 (43.5)	0.038
Age (years)	13.2 ± 4.8	11.46 ± 5.17	15.50 ± 3.24	0.002
Age group (years)				
0–9		10 (26.3)	2 (4.8)	0.001
10–19		16 (42.1)	18 (42.9)	0.612
Symptoms				
Fever	34 (73.9)	24 (92.3)	10 (50.0)	0.001
Tender lymph node	34 (73.9)	20 (76.9)	14 (70.0)	0.081
Weight loss (>2 kg)	4 (8.7)	2 (7.7)	2 (10.0)	0.127
Arthralgia	4 (8.7)	2 (7.7)	2 (10.0)	0.127
Rash	6 (13.0)	3 (11.5)	3 (15.0)	0.091
Lymphadenopathy				
Cervical (%)	44 (95.7)	24 (92.3)	18 (90.0)	0.211
Unilateral (%)	26 (56.5)	12 (46.2)	14 (70.0)	0.002
Bilateral (%)	18 (39.1)	14 (53.8)	4 (20.0)	0.002
Generalized (%)	2 (4.3)	1 (1.4)	1 (5.0)	0.081
LN max. diameter (cm)	2.35 ± 0.57	2.38 ± 0.51	2.30 ± 0.67	0.078
Relapse	4 (8.7)	4 (15.8)	0 (0.0)	0.001
Laboratory findings				
WBC (/mm^3^)	4,165 ± 1,366	4,482 ± 1,484	3,690 ± 1,083	0.098
ANC (/mm^3^)	2,110 ± 1,017	2,137 ± 1,202	2,069 ± 731	0.199
Hb (mg/dL)	12.9 ± 2.4	13.1 ± 9.3	12.8 ± 7.3	0.285
Platelet (/mm^3^)	266 ± 71	169 ± 91	299 ± 93	0.022
LDH (IU/l)	509 ± 340	602 ± 407	370 ± 139	0.061
AST (IU/l)	53 ± 46	68 ± 54	32 ± 13	0.062
ALT (IU/l)	42 ± 45	54 ± 54	23 ± 11	0.078
CRP (mg/dl)	0.93 ± 0.98	1.31 ± 1.13	0.40 ± 0.32	0.032
EBV positive, n/N (%)	8/38 (22.2)	6/16 (37.5)	2/12 (16.7)	0.025
ANA positive, n/N (%)	7/30 (26.6)	6/20 (30.0)	1/10 (10.0)	0.021

*N indicates the total number of patients who underwent the test*.

*Data are shown as mean ± standard deviation or median with interquartile range or percentage*.

*LN, lymph node; WBC, white blood cell; ANC, absolute neutrophil; Hb, hemoglobin; LDH, lactate dehydrogenase; AST, aspartate transaminase; ALT, alanine transami-nase; CRP, C-reactive protein; EBV, Epstein-barr virus; ANA, anti-nuclear antibodies*.

In adults, the female sex ratio was higher, especially for patients aged 20–29 years (Table 3). Tender lymph nodes were more common in women than in men (54.5 vs. 16.7%, *P* = 0.001). Unilateral cervical lymphadenopathy was observed more frequently in women than in men (63.6 vs. 33.3%, *P* = 0.007). The recurrence rate was also significantly higher in women than in men (18.2 vs. 0.0%, *P* = 0.005). ANA positivity rates were higher in women than in men (Table 3).

**Table 3:** Manifestations of Kikuchi-Fujimoto disease in adults by sex.

**Characteristics**	**Total**	**Male**	**Female**	***P*-value**
Number of patients (%)	34 (100)	12 (35.3)	22 (64.7)	0.018
Age (years)	32.71 ± 8.82	32.50 ± 8.02	32.82 ± 9.60	0.459
Age group (years)				
20–29	14 (17.5)	4 (10.5)	10 (23.8)	0.011
30–39	14 (17.5)	6 (15.8)	8 (19.0)	0.109
>40	6 (7.5)	2 (5.3)	4 (9.5)	0.187
Symptoms				
Fever	18 (52.9)	6 (50.0)	12 (54.5)	0.214
Tender lymph node	14 (41.2)	2 (16.7)	12 (54.5)	0.001
Weight loss (>2 kg)	6 (17.6)	2 (16.7)	4 (18.2)	0.127
Arthralgia	4 (11.8)	1 (8.4)	3 (13.6)	0.127
Rash	0 (0.0)	0 (0.0)	0 (0.0)	0.091
Lymphadenopathy				
Cervical (%)	28 (82.4)	10 (83.3)	18 (81.8)	0.383
Unilateral (%)	18 (52.9)	4 (33.3)	14 (63.6)	0.007
Bilateral (%)	10 (29.4)	6 (50.0)	4 (18.2)	0.013
Generalized (%)	6 (17.6)	2 (16.7)	4 (18.2)	0.456
LN max. diameter (cm)	1.93 ± 0.46	1.40 ± 0.45	1.22 ± 0.38	0.203
Relapse	4 (11.8)	0 (0.0)	4 (18.2)	0.005
Laboratory findings				
WBC (/mm^3^)	4,333 ± 2,264	4,173 ± 1,348	4,453 ± 2,860	0.521
ANC (/mm^3^)	2,845 ± 1,916	2,745 ± 1,207	2,920 ± 2,401	0.365
Hb (mg/dL)	12.7 ± 3.1	12.8 ± 2.4	12.6 ± 3.4	0.771
Platelet (/mm^3^)	251 ± 89	270 ± 86	239 ± 92	0.214
LDH (IU/l)	416 ± 349	354 ± 74	454 ± 453	0.337
AST (IU/l)	42 ± 33	40 ± 22	44 ± 41	0.451
ALT (IU/l)	37 ± 74	20 ± 7	49 ± 99	0.468
CRP (mg/dl)	1.82 ± 2.94	1.94 ± 2.27	1.72 ± 3.52	0.399
EBV positive n/N (%)	0/10 (0.0)	0/3 (0.0)	0/7 (0.0)	NA
ANA positive, n/N (%)	7/16 (43.7)	2/6 (33.3)	5/10 (50.0)	0.037

*N indicates the total number of patients who underwent the test*.

*Data are shown as mean ± standard deviation or median with interquartile range or percentage*.

*LN, lymph node; WBC, white blood cell; ANC, absolute neutrophil; Hb, hemoglobin; LDH, lactate dehydrogenase; AST, aspartate transaminase; ALT, alanine transami-nase; CRP, C-reactive protein; EBV, Epstein-barr virus; NA, not available; ANA, anti-nuclear antibodies*.

Most patients received non-steroidal anti-inflammatory drugs (NSAIDs) as a first-line treatment for KFD. Ibuprofen (30–40 mg/kg in three divided doses) was given in most young children and naproxen (10–20 mg/kg, two divided doses, max 1,000 mg/day) in older children and adults. Corticosteroid treatment was added if the fever did not improve even 2–5 days after starting the NSAIDs. The usual dose of oral corticosteroid (prednisolone) was 1 mg/kg (max 60 mg) in three divided and used for 3–10 days in 15 adults and 18 children. There were no patients requiring different therapies other than oral steroids and NSAIDs. In some patients (11 adults and 8 children), the symptoms were improved without any specific treatment. In the case of relapse, treatment was performed at each time of relapse in a similar way.

The clinical characteristics of the patients with Kikuchi disease recurrence are described in Table 4. There were four children and four adults. All children were male, while all adults were female. The range of fever duration was 9–30 days. Two children were EBV-positive. ANA positive results were observed in two children and three adults.

**Table 4:** Kikuchi-Fujimoto disease patients with relapse.

**No**	**Age (years)**	**Gender**	**Fever duration (days)**	**LN site**	**LN maximum size (cm)**	**EBV positive**	**ANA positive**	**No of relapses**	**Follow- up duration (years)**
1	6	M	16	Unilateral C. and involved inguinal	2.5	+	–	1	3
2	7	M	14	Unilateral C.	1.5	–	–	1	2
3	8	M	9	Bilateral C.	2.0	–	+	2	4
4	8	M	14	Unilateral C	2.5	+	+	1	1
5	29	F	30	Unilateral C. and Involved axilla	2.0	–	+	2	3
6	32	F	30	Involved axilla	1.5	–	–	1	2
7	38	F	15	Unilateral C. and Involved axilla	1.0	–	+	1	4
8	47	F	20	Bilateral C.	2.0	–	+	3	6

*LN, lymph node;. EBV, Epstein-barr virus; ANA, anti-nuclear antibodies; C, cervical*.

## Discussion

KFD has a broad clinical spectrum, including fever and cervical lymphadenopathy, the most common symptoms. Typically, patients with KFD present clinically as fever and posterior cervical lymphadenopathy. These patients can have leukopenia as a clue. In our study, we investigated the clinical and laboratory characteristics and KFD according to age and sex. We found that there were some differences in clinical patterns with age. The prevalence of KFD is known to be higher in Asian and Eastern European populations (1, 3, 14). KFD usually occurs in the 30s and 40s and has a female predominance with a ratio of ~3–4:1 in young adults (15); however, in children, there are inconsistent results in terms of sex differences, although it seems that female predominance, as seen in young adults, is not evident (2, 6, 16–18). Moreover, some studies reported male predominance in children (2, 17, 18). Kim et al. showed a male predominance under the age of 14 (1:1.6) and a female predominance over the age of 15 (3:1) (2). This difference may be related to race and the small number of pediatric patients in the studies. To the best of our knowledge, there have been few papers comparing adults and children in one institution, and our study has the advantage of being performed under controlling the variables of race and region. We found male predominance among children and female predominance among adults in one institution, and these differences were statistically significant. Although the exact pathophysiology of KFD is still unknown, it may be assumed that there are different pathological mechanisms in children compared to those in adults. The youngest child was 3 years old, with a mean age of 13.2 years, similar to previous studies (6, 15, 17).

We identified differences in symptoms and laboratory findings between children and adults. Fever, tenderness in the lymph node, and skin rashes were more common in children, while myalgia and weight loss were more common in adults, with more systemic inflammatory symptoms in children than in adults. Lymphadenopathy was less common in children. These results are almost consistent with those of previous studies by Kim et al. (2). Although it is not clear why there were more systemic symptoms in children, we suspect that differences in immune responses between children and adults, roles of sex hormones, and infection frequencies may play a role; however, further research is needed. In a study comparing clinical-cytological features in adults and children, children were significantly less likely to have high cellularity and Kikuchi histiocyte counts >5% than adults (19).

Our study also showed that the positive rate of EBV, one of the trigger factors of KFD, was higher in children, particularly among boys compared to adults. Although ANA positivity was higher in women than in men, ANA positivity was higher in boys than in girls. ANA-positive findings in boys are suspected to be related to EBV infection rather than persistent autoimmunity. EBV during the acute infection or reactivation phase could lead to the formation of the ANA and extractable nuclear antigen autoantibodies (ENA) (20). Although there were no patients with overt systemic lupus erythematosus (SLE) during the study period, careful observation and follow-ups are required due to the fact that autoimmunity due to EBV infection could be related to the development of SLE (21, 22).

SLE is a close differential diagnosis in patients with KFD. We should be careful if SLE occurs during follow-up, especially in the case of positive ANA, because some patients with KFD have associated lupus or develop lupus on follow-up (1.3~25%) (23, 24). Monogenic lupus is a kind of SLE that generally presents early in life, usually at <5 years of age. Recently, a significant number of genes have been implicated in monogenic lupus, such as several complement deficiencies, *ACP5, DNASE1, DNASE1L3, PRKCD, RAG2* genes, etc. (25). An interesting association between C1q deficiency lupus with Kikuchi-Fujimoto disease and macrophage activation syndrome was reported (26). C1q deficiency is a rare cause of early-onset SLE. As in typical SLE, KFD can also occur in early-onset SLE due to complement deficiency such as C1q deficiency.

KFD is generally known to be self-limiting local lymphadenopathy; however, pediatricians sometimes experience recurrent or refractory KFD in children. Although the recurrence rate of KFD is usually known to be around 3–4%, it has a higher recurrence rate in children (3, 27, 28). Yoo et al., in a multivariate analysis, reported a 42% recurrence rate in 33 children with KFD; they suggested that a past history of systemic illnesses and a higher absolute lymphocyte count were risk factors associated with recurrent KFD (9). This study may have shown a slightly higher rate of recurrence because it included patients who had relapsed before the KFD was confirmed. In our study, the total relapse rate was 10%; interestingly, all relapsed patients were male in children, while all were female in the adult group. In a recent study of 98 children with KFD, there was a higher proportion of boys who had recurrent KFD, although this was not statistically significant (6). We ruled out relapsed patients before the KFD was confirmed because we were not sure whether it was a definite symptom of KFD.

Corticosteroids are commonly used for treatment. Patients with frequent recurrences are likely to suffer from side effects of corticosteroids; thus, they usually require the administration of steroid-sparing drugs instead of long-term use corticosteroids, as in other autoimmune diseases. In KFD, hydroxychloroquine is known to be effective and can be an alternative to corticosteroids because of its favorable effects and safety (29–31). Hydroxychloroquine, developed initially as an antimalarial drug, is commonly used in the treatment of rheumatic diseases such as systemic lupus erythematosus and dermatomyositis due to its immunomodulatory effects (32–34). Hydroxychloroquine suppresses the production of proinflammatory cytokines produced by peripheral mononuclear cells in the blood, such as IFNγ, TNFα interleukin (IL)-1, and IL-6 (27, 35, 36). Impaired apoptosis of self-reactive effector T cells is an essential mechanism for autoimmunity. Hydroxychloroquine can suppress autoimmunity by promoting apoptosis in effector T cells and inhibiting T cell antigen receptor signaling (37, 38). In KFD, hydroxychloroquine can be effective for the resolution of fever and systemic symptoms by impairing the production of IFN-γ (30, 39). In our study, there were no patients who took hydroxychloroquine because there were no cases of steroid dependence or refractory, and patients with multiple recurrences were rare (only one patient with three relapses, The interval between each relapse was about 1–2 years).

This study has some limitations. First, this was a retrospective study with a relatively small number of patients in a single hospital. Second, this was not a cohort study, so we could not fully understand the patients' current conditions. Third, we described the pathologic findings (description only). However, finer pathologic details of individual cases were not included since the focus of the manuscript was on clinical characteristics. In conclusion, our findings that suggest evident differences in the clinical and laboratory features of KFD according to age are encouraging. Ideally, our results aid in improving our understanding of KFD according to age and sex and are helpful for clinicians.

## Data Availability Statement

The raw data supporting the conclusions of this article will be made available by the authors, without undue reservation.

## Ethics Statement

The studies involving human participants were reviewed and approved by the Institutional Review Board of Pusan National University Hospital, Busan, Korea (11 January 2021; protocol code 2012-032-098). Written informed consent from the participants' legal guardian/next of kin was not required to participate in this study in accordance with the national legislation and the institutional requirements.

## Author Contributions

H-YK, HJ, and SK contributed to conception and design of the study and wrote sections of the manuscript. HJ organized the database. SK performed the statistical analysis. H-YK and SK wrote the first draft of the manuscript. All authors contributed to manuscript revision, read, and approved the submitted version.

## Conflict of Interest

The authors declare that the research was conducted in the absence of any commercial or financial relationships that could be construed as a potential conflict of interest.

## Publisher's Note

All claims expressed in this article are solely those of the authors and do not necessarily represent those of their affiliated organizations, or those of the publisher, the editors and the reviewers. Any product that may be evaluated in this article, or claim that may be made by its manufacturer, is not guaranteed or endorsed by the publisher.
